# Antimicrobial Stewardship Program for Patients in the Hematological Department Receiving Carbapenem Therapy: A Single-Center and Interrupted Time Series Analysis

**DOI:** 10.3390/antibiotics12020302

**Published:** 2023-02-02

**Authors:** Ayako Suzuki, Fumihiro Yamaguchi, Masayuki Maeda, Miyuki Hashiguchi, Nobuyuki Kabasawa, Jun Sasaki, Tokutada Sato, Masaki Fuyama, Yohei Yamazaki, Kei Endo, Kae Iwata, Sei Kobayashi, Hisato Fujihara

**Affiliations:** 1 Department of Pharmacy, Showa University Fujigaoka Hospital, Yokohama 227-8501, Japan; 2 Antimicrobial Stewardship Team, Showa University Fujigaoka Hospital, Yokohama 227-8501, Japan; 3 Department of Respiratory Medicine, Showa University Fujigaoka Hospital, Yokohama 227-8501, Japan; 4 Division of Infection Control Sciences, Department of Clinical Pharmacy, Showa University School of Pharmacy, Tokyo 142-8555, Japan; 5 Department of Nursing, Showa University Fujigaoka Hospital, Yokohama 227-8501, Japan; 6 Department of Hematology, Showa University Fujigaoka Hospital, Yokohama 227-8501, Japan; 7 Department of Emergency and Critical Care Medicine, Showa University Fujigaoka Hospital, Yokohama 227-8501, Japan; 8 Department of Cardiology, Showa University Fujigaoka Hospital, Yokohama 227-8501, Japan; 9 Department of Pediatrics, Showa University Fujigaoka Hospital, Yokohama 227-8501, Japan; 10 Department of Diabetes Metabolism and Endocrinology, Showa University Fujigaoka Hospital, Yokohama 227-8501, Japan; 11 Department of Inspection, Showa University Fujigaoka Hospital, Yokohama 227-8501, Japan; 12 Department of Otorhinolaryngology, Showa University Fujigaoka Hospital, Yokohama 227-8501, Japan; 13 Department of Hospital Pharmaceutics School of Pharmaceutical Sciences, Showa University, Tokyo 142-8666, Japan

**Keywords:** antimicrobial stewardship, hematological disorders, carbapenem, interrupted time series analysis, predictive factors

## Abstract

As antibiotic resistance has become a global problem, the intervention of an antimicrobial stewardship team (AST) is warranted. In hematological disorders, infectious complications are crucial owing to abnormal neutrophil function and decreased cell-mediated immunity. Despite the widespread implementation of AST intervention, the effectiveness of stewardship practices for immunocompromised patients remains uncertain. We determined the effect of AST interventions on carbapenem therapy in the department of hematology. Patients admitted to the department and undergoing carbapenem therapy were enrolled. We compared carbapenem use between the pre-AST (April 2016–March 2018) and post-AST (April 2018–March 2021) periods. Factors associated with long-term carbapenem therapy were investigated. Overall, 515 episodes of carbapenem therapy in 264 patients in the department were evaluated. According to the interrupted time series analysis, the number of days of therapy decreased with AST intervention (β = −0.263, *p* = 0.011). In multivariate analysis, predictive factors associated with long-term carbapenem therapy (>8 days) were outpatient onset, chronic obstructive pulmonary disease, acute myeloid leukemia, multiple myeloma, and infection with resistant bacteria (such as extended spectrum β-lactamases and AmpC) (95% confidence interval, 1.030–2.818, 1.067–66.667, 1.057–2.782, 0.168–0.742, and 1.382–5.750, respectively). The AST intervention reduced carbapenem use in patients with hematological disorders.

## 1. Introduction

For patients with hematological disorders, infectious complications are crucial owing to the occurrence of abnormal neutrophil function and decreased cell-mediated immunity [1]. In such patients, neutropenia is frequently induced by chemotherapy or radiotherapy. Notably, the mortality rate of febrile neutropenia (FN) has been reported to be 2–20% [2], and the clinical guidelines for FN recommend to immediately initiate empirical therapy of broad-spectrum antibiotics, including carbapenems [1,3,4,5]. The long-term use and broad-spectrum antibiotic exposure not only lead to side effects, such as allergies and kidney or liver toxicity, but also infections caused by antimicrobial-resistant bacteria.

Antimicrobial stewardship programs (ASPs) have been identified as a valuable tool for optimizing antibiotic therapy, and these programs involve the promotion of the de-escalation strategy, post-prescription review and feedback (PPRF) strategy, and clinical education [6]. Moreover, a carbapenem-sparing strategy has been recommended to conserve the efficacy of carbapenems and reduce drug-resistant gram-negative bacteria [7,8]. Although ASPs have been implemented in many medical institutions, specific data on the efficacy of ASPs practices in patients with immunocompromised status remain unclear. European guidelines have indicated that empirical antibiotics can be discontinued in patients who have been afebrile for ≥48 h, regardless of their neutrophil count or expected duration of neutropenia [3]. However, a voluntary survey in the USA reported apparent issues associated with antimicrobial stewardship, including undefined duration for certain infections, perception that antibiotic-resistant infections require de-escalation (DE), and prescriber opposition [9]. Thus, the selection and discontinuation of antimicrobial agents should be conducted following comprehensive consideration of clinical expertise, patient status, and scientific evidence.

We established an antimicrobial stewardship team (AST) and collaborated with hematologists to promote the appropriate antibiotic use of antibiotics. This study aimed to determine the effect of AST intervention on carbapenem stewardship for patients in the department of hematology. Furthermore, as factors contributing to long-term carbapenem therapy in patients with hematological disorders have not been clarified to date, we investigated the factors associated with long-term carbapenem therapy.

## 2. Results

### 2.1. Characteristics of the Participants

**Figure 1:**
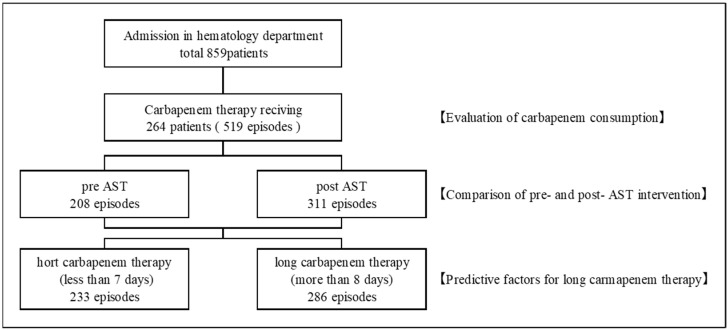
Patient information. Many patients were repeatedly hospitalized. Among them, carbapenems were used multiple times, and we evaluated each use.

Overall, 264 patients were enrolled in this study (Figure 1). The clinical characteristics of these patients are summarized in Table 1. The age of the patients ranged from 24.0 to 94.0 years, and there were 157 (59.5%) men and 107 (40.5%) women in the study. Moreover, 89 (33.7%), 31 (11.7%), and 7 (2.7%) patients had chronic kidney disease (CKD), diabetes mellitus, and chronic obstructive pulmonary disease (COPD), respectively. In addition, 77 (29.2%) and 84 (31.8%) patients had acute myeloid leukemia and malignant lymphoma, respectively.

Table 1 shows the patient background in the pre- and post-AST periods. During the study period, 519 episodes were recorded, including 208 and 311 in the pre- and post-AST periods, respectively. All results were followed by pre- and post-AST, respectively. The median ages of the participants in the pre- and post-AST periods were 71.0 and 71.0 years, respectively. Notably, men accounted for 61.5% and 53.7% of the participants in the pre- and post-AST periods, respectively. Both groups had low body mass index (pre-AST vs. post-AST; 19.8 vs. 20.9 kg/m^2^). Furthermore, the CKD rates were 46.2% and 43.4% in the pre- and post-AST periods, respectively.

At the initiation of carbapenem therapy, the number of granulocytes (pre-AST vs. post-AST; 434.5 vs. 216.0/µL) and body temperature (pre-AST vs. post-AST; 38.0 vs. 38.1 °C) were also comparable between the two groups. Notably, the FN rates were 47.1% and 55.9% in the pre- and post-AST periods, respectively, because a larger proportion of patients received chemotherapy within 3 weeks (pre-AST vs. post-AST; 54.3% vs. 73.3%, Table S1). Moreover, 56.3% and 45.7% of patients had a fever of unknown origin (FUO), whereas 13.0% and 25.4% had a microbiologically documented infection (MDI) in the pre- and post-AST periods, respectively (Table S1).

The incidence of hematological disorders is shown in Table 1. Regarding the type of carbapenem used, 56.3% and 78.5% of patients received meropenem in the pre- and post-AST periods, respectively. Regarding the medical devices used, 23.6% and 57.9% of the patients used central venous catheters in the pre- and post-AST periods, respectively. Other clinical data about disease, medication, and medical device are presented in Table S1.

### 2.2. Carbapenem Consumption

**Figure 2:**
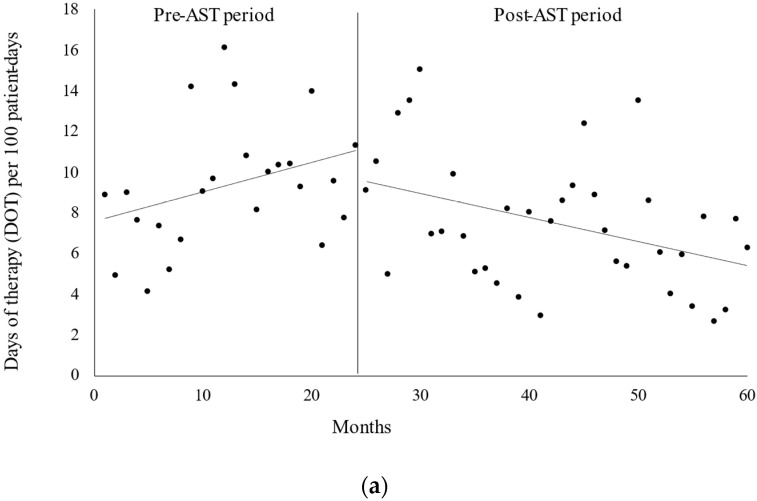

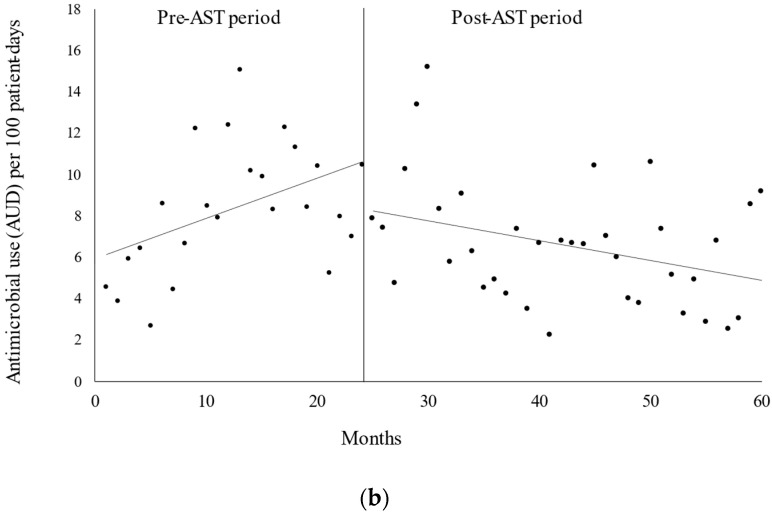
(**a**) Interrupted time series analysis of the trends in days of therapy (DOT) for carbapenem per 100 patient-days evaluated before and after the implementation of the antimicrobial stewardship team (AST). Solid lines indicate the observed trend in the pre- and postimplementation periods. After AST implementation, the trend in the monthly DOT decreased (β = −0.263, *p* = 0.011). (**b**) Interrupted time series analysis of the trends in carbapenem antimicrobial use density (AUD) per 100 patient-days evaluated before and after the implementation of the antimicrobial stewardship program. Solid lines indicate the observed trend in the pre- and postimplementation periods. After AST implementation, the trend in the monthly AUD decreased (β = −0.291, *p* = 0.003).

The interrupted time series analysis (ITSA) revealed a sustained reduction in favor of the expected antimicrobial consumption based on the pre-intervention trend (Figure 2). The trend in the number of days of therapy (DOT) for carbapenem was observed during the pre-AST period (β1 = 0.146, *p* = 0.101), and after the AST intervention, the level (β2 = −1.681, *p* = 0.292) was not changed and the slope decreased (β3 = −0.263, *p* = 0.011) (Figure 2a). Moreover, the trend in antimicrobial use density (AUD) for carbapenem increased during the pre-AST period (β1 = 0.195, *p* = 0.023), and after the AST intervention, both the level (β2 = −2.566, *p* = 0.094) and slope decreased (β3 = −0.291, *p* = 0.003) (Figure 2b).

Changes in other antibiotics, including tazobactam/piperacillin, fourth-generation cephalosporins, and third-generation cephalosporins, were shown in Table 2.

### 2.3. Comparison between Pre-AST and Post-AST Groups

Table 3 shows the effects of AST implementation. The number of ≥2 sets of blood cultures collected was significantly higher in the post-AST period than in the pre-AST period (96 episodes vs. 268 episodes, *p* < 0.001). Moreover, the blood culture positivity cases during the investigation period increased in the post-AST period (36 episodes vs. 89 episodes, *p* = 0.003). The positive rate of blood cultures was similar between the pre- and post-AST periods (37.5% vs. 33.2%, *p* = 0.447). In addition, the number of nursing records regarding the evaluation of safety in the initial administration of antimicrobials increased in the post-AST period (3.4% vs. 10.6%, *p* = 0.002). Notably, the duration of carbapenem therapy did not differ between the pre-AST and post-AST periods (8.0 vs. 8.0 days, *p* = 0.542). The number of days in which carbapenem therapy where <500 granulocytes was not achieved was administered was significantly longer in the post-AST than in the pre-AST period (0.0 vs. 3.0 days, *p* < 0.001). No difference was noted in the 30-day mortality rate (21.2% vs. 19.0%, *p* = 0.541) or the in-hospital mortality rate (27.9% vs. 26.4%, *p* = 0.703). Other effects of AST implementation on biological changes or carbapenem perception trends in the pre- and post-AST periods are shown in Table S2. Furthermore, the percentage of gram-positive cocci detected in blood cultures increased in the post-AST period (7.2% vs. 17.4%, *p* = 0.001). However, within the first 7 days of treatment, the rate of discontinuation (28.4% vs. 30.2%, *p* = 0.649), change to narrow spectrum agents as beta-lactam levels (7.2% vs. 8.7%, *p* = 0.547), and the targeted therapy dose (1.0% vs. 3.5%, *p* = 0.086) remained unchanged (Table S2).

### 2.4. Predictive Factors for Long-Term Carbapenem Therapy

In univariate analysis, we compared 286 episodes of long-term carbapenem therapy (>8 days) with 233 episodes of short-term carbapenem therapy (<7 days) (Table 4 and Table S3). Notably, patients in the long-term carbapenem therapy group were younger than those in the short-term carbapenem therapy group (72.0 vs. 70.0 years, *p* = 0.049). Moreover, granulocyte counts were lower in the long-term carbapenem therapy group than in the short-term carbapenem therapy group (456.0 vs. 162.0/µL, *p* = 0.005). In addition, the rate of outpatient incidence using carbapenems during hospitalization was higher in the long-term carbapenem therapy group than in the short-term carbapenem therapy group (14.2% vs. 21.0%, *p* = 0.044). Furthermore, the comorbidity of diabetes mellitus (8.2% vs. 13.6%, *p* = 0.049) and COPD (0.4% vs. 4.9%, *p* = 0.002) were associated with the long-term carbapenem therapy. The frequency of usage of indwelling urinary catheters as medical devices was higher in the long-term carbapenem therapy group than in the short-term carbapenem therapy group (30.9% vs. 20.3%, *p* = 0.005). The incidence of FUO was lower in the long-term carbapenem therapy group than in the short-term carbapenem therapy group (55.8% vs. 45.1%, *p* = 0.015). However, clinically documented infection (CDI) was more common in the long-term carbapenem therapy group than in the short-term carbapenem therapy group (24.9% vs. 33.6%, *p* = 0.031). In addition, episodes with acute myeloid leukemia were more likely to receive long-term carbapenem therapy (23.6% vs. 41.3%, *p* < 0.001), whereas episodes with malignant lymphoma (35.2% vs. 25.5%, *p* = 0.017) and multiple myeloma (12.0% vs. 4.9%, *p* = 0.003) were more likely to receive short-term carbapenem therapy. The rate of detection of infection with resistant bacteria, including extended spectrum beta-lactamase (ESBL) or AmpC, was higher in the long-term carbapenem therapy group than in the short-term carbapenem therapy group (6.9% vs. 68.9%, *p* = 0.023).

Predictive factors associated with long-term carbapenem therapy in the multivariate analysis are shown in Table 5. Outpatient onset, COPD, acute myeloid leukemia, and infection with resistant bacteria (ESBL and AmpC) were associated with long-term carbapenem therapy (odds ratio [OR] = 1.704, 8.436, 1.715, and 2.819, 95% confidence interval [CI] = 1.030–2.818, 1.067–66.667, 1.057–2.782, and 1.382–5.750, respectively), whereas multiple myeloma was associated with short-term carbapenem therapy (OR = 0.364, CI = 0.168–0.792).

## 3. Discussion

This study revealed that AST intervention in patients with hematological disorders decreased carbapenem use and increased the collection of blood cultures based on comparing pre- and post-AST periods. In addition, the predictive factors for long-term carbapenem therapy (>8 days) were outpatient onset, COPD, acute myeloid leukemia, detection of resistant bacteria (such as ESBL and AmpC), and multiple myeloma.

Notably, AST intervention reduced the DOT and AUD of carbapenem in patients with hematological disorders. Several studies have reported that AST intervention reduces carbapenem use based on the relevant guidelines, and the appropriate use of carbapenems in hematology is a major concern [10,11,12,13,14,15,16,17]. However, in some cases, the attending physicians could not comply with the guidelines. Moreover, owing to the complexity of the disease, carbapenems have not been widely adopted [9].

The AST intervention facilitated the increase in the collection of blood cultures (>2 sets). In general, blood culture examinations should be performed during antimicrobial therapy for patients with FN [4,18]. Moreover, two sets of blood cultures should be collected during the treatment of infectious diseases even if the patient has a hematological disorder.

In addition, we reduced the duration of carbapenem therapy with <500 granulocytes. Notably, the ANTIBIOSTOP study indicated that adherence to the latest European Conference on Infections in Leukemia (ECIL)-4 guidelines would reduce the duration of empirical therapy [19]. The need for discontinuation of carbapenem therapy in patients without fever and with a granulocyte count of <500 has been discussed in the relevant literature. However, hematologists assess that early discontinuation is dangerous. Therefore, AST interventions were acceptable to them, and they carefully determined whether the therapy could be implemented. In the present study, carbapenem use was found to be decreased despite the fact that the administration period did not change because of the decrease in the choice of carbapenems used.

We instructed the prescribing behavior of hematologists for carbapenem therapy without strict criteria including body temperature, neutrophil, bacteriological results, or therapy duration. Notably, we responded flexibly to prescribing behavior for patient-oriented medical care [20,21]. Accordingly, ASPs are shared with the treatment plan for infectious diseases by PPRF, and the attending physicians makes the appropriate decisions for infectious diseases, which further increases the educational effect [6]. As such, ASTs and hematologists need to learn each other’s fields.

We suggest that these results are attributed to the following factors. Our AST shared the policy and adopted the PPRF strategy; accordingly, the hematologist collected two sets of blood cultures before the carbapenem therapy. Several blood cultures results revealed bacterial infections, which resulted in the discontinuation of carbapenem therapy with <500 granulocytes. Subsequently, we appropriately reduced the DOT and AUD of carbapenems.

To the best of our knowledge, this is the first study to reveal the predictive factors for long-term carbapenem therapy in hematological disorders. Understanding of factors contributing to long-term dosing is useful for ascertaining guidelines for treatment. In the present study, a trend toward long-term administration of carbapenem was noted in patients with acute myeloid leukemia, whereas the trend was toward short-term administration occurred in those with multiple myeloma. Generally, acute myeloid leukemia is associated with a worse prognosis, whereas multiple myeloma is associated with a better prognosis [22,23,24,25,26]. As patients with acute myeloid leukemia have a poor prognosis and develop neutropenia, they need long-term carbapenem therapy; indeed, the trends of carbapenem duration were different for each hematological disease. In the multivariate analysis, an outpatient onset was significantly associated with long-term carbapenem therapy. In contrast to our expectations, based on the Multinational Association of Supportive Care in Cancer score, outpatient onset is associated with low risk [27,28]. In general, low-risk FN can be followed-up in an outpatient setting, but carbapenem use during hospitalization was found to be prolonged. Our hospital is a tertiary emergency medical center and is well equipped to safely manage patients with FN in the emergency room during outpatient management.

Infection is an exacerbating factor of COPD, and it is likely to occur because innate lung defenses are impaired in cases of COPD [29]. In the present study, the overlap of COPD with FN as a factor involved in the exacerbation of infection was found to be a high-risk factor, suggesting that COPD should be carefully examined. Moreover, the detection of resistant organisms was identified as a factor associated with prolonged treatment. When resistant organisms, such as ESBL, are detected, carbapenem therapy is often pursued as a targeted therapy, and the standard treatment period for gram-negative bacilli with positive results based on blood culture is approximately 7 days [30,31], although it is often necessary to prolong therapy for patients with immunosuppressed status.

This study has several limitations. First, the retrospective design of this before–after and single-center study may have resulted in immeasurable confounders, which were not adjusted; moreover, we were unable to evaluate adherence to interventions. Second, because autologous hematopoietic stem cell transplantation has increased, patients in the post-AST cohort might become more seriously ill. This may have led to more severe morbidity in the post-AST period. However, this study reported a decrease in carbapenem consumption and no 30-day mortality. Third, in 2019, there was a significant shortage of various antimicrobials other than carbapenems in Japan [32]. The evaluation of the AST intervention instead of the promotion of DE during this period was challenging. Fourth, regardless of appropriate culture testing, resistant bacteria were rarely detected, and changes in the resistance rate could not be examined.

## 4. Materials and Methods

### 4.1. Population and Data Collection

This comparative study was conducted according to ASPs from April 2016 to March 2021 at Showa University Fujigaoka Hospital. The study population included all inpatients in the department of hematology who received carbapenems during the study periods. The pre-implementation period was from April 2016 to March 2018, whereas the postimplementation period was from April 2018 to March 2021.

The use of carbapenems and clinical outcomes were compared before and after ASP intervention. Moreover, data regarding antibiotic use were collected for each inpatient day. The AUD was calculated according to the following formula: AUD of antibiotics = defined daily dose (DDD) of antibiotics per 100 patient-days in the department of hematology. The DDD was considered as an average maintenance dose per day of the corresponding antibiotic used for its main indication in adults [33]. The DOT was defined as any dose of antibiotics administered per 100 patient-days. All clinical data were obtained from the medical records of patients receiving each carbapenem therapy.

Notably, the eGFR of each patient was calculated using the following formula: eGFR (mL/min/1.73 m^2^) = 194 × serum creatinine^−1.094^ × age^−0.287^ × 0.739 (female) [34]. The CKD was defined as the eGFR of <60 mL/min/1.73 m^2^, as within the G3–G5 levels, as per the Kidney Disease: Improving Global Outcomes (KDIGO) guidelines [35]. Notably, long-term carbapenem therapy was defined by the duration of >8 days. Previously, we investigated the trend of carbapenem prescription include DE within 7 days of prescribing given that blood culture test results can be analyzed for approximately 1 week [36].

The effect of AST intervention was evaluated by blood culture collection, detected bacteria, duration (days) of carbapenem therapy, prescription trends, and 30-day mortality rate. The minimum inhibitory concentrations of meropenem were determined using the broth microdilution method and were interpreted according to the Clinical and Laboratory Standards Institute document M100-S22. These clinical data were used to investigate the factors that led to the requirement of carbapenem therapy for >8 days. According to the clinical evaluation and microbiological results, the patients were classified as having FUO, MDI, or CDI. Here, CDI referred to episodes where an infection site could be identified—clinically or radiologically—without microbiological documentation.

The study protocol was approved by the Institutional Ethics Committee of Showa University (approval number: 21-092-B). The requirement of obtaining informed consent from the patients was waived owing to the retrospective nature of this study.

### 4.2. AST Implementations

The AST comprised physicians who belonged (a total full equivalent, FTE = 0.01) to the respiratory, emergency, cardiology, gastroenterology, endocrinology, hematology, or pediatrics department; a pharmacist (FTE = 0.1); infection control nurses (FTE = 0.02); and a microbiology technologist (FTE = 0.02). The AST conferences for patients receiving carbapenems were held once in a week in a conference room. Before the establishment of the AST, no intervention strategy of antimicrobial stewardship against any prescription including carbapenems was established at our institution. The aim of the PPRF strategy was to optimize the use of antimicrobials. These interventions encouraged physicians to determine appropriate antibiotics, dosage amount, and duration by performing microbial examination including blood culture tests. The AST provided recommendations to physicians based on the data obtained from the medical records. If the physicians did not follow the recommendations after 3–5 days of the intervention, AST members discussed the antimicrobial therapy with them and provided secondary recommendations.

### 4.3. AST Policy against the Hematology Department

The policies for against the hematological department were defined as follows: (1) referral to the clinical guidelines for stewardship in hematology published by the ECIL group as well as practical guidelines for FN published by the Japanese Society of Medical Oncology, (2) collection of two sets of blood cultures before starting antibacterial therapy and before escalation, (3) use of broad-spectrum antibiotics by hematologists without permissions from AST, (4) use of carbapenems therapeutically and not prophylactically, (5) not performing any chemotherapy in cases of severe infections, (6) consideration of early discontinuation or DE of broad-spectrum antibiotics in cases of neutropenia without fever, (7) sharing treatment goals with AST regarding antimicrobial duration, status of the hematological disorder, and hospitalization period in case of long-term carbapenem administration, and (8) following AST recommendations for blood examination, imaging studies, and appropriate antimicrobial agents as required.

### 4.4. Statistical Analysis

The ITSA was performed to identify the effect of the intervention on the AUD and DOT of carbapenem. The monthly data points in the pre- and post-AST periods were available for the analysis. The model included an intercept (β0), baseline trend (β1), level change after the start of the intervention (β2), and trend change after initiating the intervention (β3). Data were expressed as the median (interquartile range) for continuous variables and percentages for categorical variables. The Mann–Whitney rank-sum test was used to compare mean values between the two groups. The Pearson’s chi-squared or Fisher’s exact test was used for the univariate analysis of the association between two categorical variables. A logistic regression model was used to evaluate long-term carbapenem therapy (>8 days), and the findings were presented as ORs with 95% CIs. Statistical significance was set at *p* < 0.05. All statistical analyses were performed using the IBM SPSS Statistics for Windows, version 23.0 (IBM Japan, Tokyo, Japan).

## 5. Conclusions

The PPRF strategy with the policy implemented by AST has enhanced the carbapenem stewardship for patients with hematological disorders. Further, the predictors for the long-term carbapenem therapy (>8 days) were identified.

## Figures and Tables

**Table 1:** Characteristics of the participants and clinical data in the pre- and post-AST periods.

Characteristic	Total *n* = 264 (Patients)	Pre-AST *n* = 208 (Episodes)	Post-AST *n* = 311 (Episodes)	*p*-Value
Age (years)	72.5 (24.0–94.0)	71.0 (24.0–94.0)	71.0 (30.0–90.0)	0.616
Sex (male)	157 (59.5)	128 (61.5)	167 (53.7)	0.077
BMI (kg/m^2^)	20.7 (12.9–42.1)	19.8 (12.9–40.0)	20.9 (13.9–42.1)	<0.001 *
CKD ^†^	89 (33.7)	96 (46.2)	135 (43.4)	0.537
Diabetes mellitus	31 (11.7)	35 (16.8)	23 (7.4)	0.001 *
COPD	7 (2.7)	11 (5.3)	4 (1.3)	0.013 *
Hematologic disease				
Acute myeloid leukemia	77 (29.2)	69 (33.2)	101 (32.5)	0.868
Myelodysplastic syndrome	30 (11.4)	23 (11.1)	42 (13.5)	0.409
Acute lymphocytic leukemia	10 (3.8)	3 (1.4)	20 (6.4)	0.008 *
Malignant lymphoma	84 (31.8)	64 (30.8)	91 (29.3)	0.713
Multiple myeloma	21 (8.0)	16 (7.7)	26 (8.4)	0.785
Aplastic anemia	8 (3.0)	13 (6.3)	12 (3.9)	0.212
Others	34 (12.9)	20 (9.6)	19 (6.1)	0.138

Number (%) or median (interquartile range). Abbreviations are as follows: BMI, body mass index; CKD, chronic kidney disease; COPD, chronic obstructive pulmonary disease. ^†^ CKD was defined by eGFR of <60 mL/min/1.73 m^2^ as G3–G5 level as per the KDIGO guidelines. * *p* < 0.05.

**Table 2:** Consumption of antibiotics demonstrating anti-pseudomonas aeruginosa activity.

Category	Consumption	Pre-AST	Post-AST	U-Value	*p*-Value
Carbapenem	DOT	9.2 (4.2–16.1)	7.1 (2.7–15.0)	270.0	0.015 *
	AUD	8.4 (2.7–15.1)	6.6 (2.2–15.2)	281.0	0.023 *
Tazobactam/piperacillin	DOT	44.5 (4.6–73.8)	47.4 (14.6–145.0)	509.0	0.245
	AUD	37.7 (3.7–75.6)	40.8 (10.6–127.1)	480.0	0.469
Fourth-generation cephalosporins	DOT	95.2 (40.8–139.3)	67.1 (0.0–151.2)	271.0	0.015 *
	AUD	79.3 (37.6–121.8)	27.7 (0.0–102.0)	65.0	<0.001 *
Third-generation cephalosporins	DOT	15.7 (0.0–67.8)	56.5 (0.0–155.8)	716.0	<0.001 *
	AUD	10.0 (0.0–48.6)	39.2 (0.0–94.2)	715.0	<0.001 *

Median (interquartile range). Abbreviations are as follows: AST, antimicrobial stewardship team. * *p* < 0.05.

**Table 3:** Effects of AST implementation in the pre- and post-AST periods.

Characteristic	Pre-AST *n* = 208 (Episodes)	Post-AST *n* = 311 (Episodes)	*p*-Value
Blood culture (2 sets) before starting antibiotics	96 (46.2)	268 (86.2)	<0.001 *
Blood culture positivity	36 (17.3)	89 (28.6)	0.003 *
Nursing record about antibiotics	7 (3.4)	33 (10.6)	0.002 *
Length of carbapenem therapy	8 (1.0–39.0)	8 (1.0–31.0)	0.542
Number of days not receiving carbapenem with <500 granulocytes	0 (0.0–180)	3 (0.0–431)	<0.001 *
30-day mortality	44 (21.2)	59 (19.0)	0.541
In-hospital mortality	58 (27.9)	82 (26.4)	0.703
Length of hospital stay	20.5 (1.0–228.0)	20.0 (1.0–383.0)	0.269
Gram-positive cocci	15 (7.2)	54 (17.4)	0.001 *

Number (%) or median (interquartile range). Abbreviations are as follows: AST, antimicrobial stewardship team. * *p* < 0.05.

**Table 4:** Univariate analysis for long-term carbapenem therapy (>8 days).

Variable	Short-Term Carbapenem Therapy (<7 Days) *n* = 233 (Episodes)	Long-Term Carbapenem Therapy (>8 Days) *n* = 286 (Episodes)	*p*-Value
AST intervention	137 (58.8)	174 (60.8)	0.637
Age	72.0 (24.0–94.0)	70 (24.0–92.0)	0.049 *
eGFR (mL/min/1.73 m^2^)	61.1 (5.4–318.0)	68.3 (3.7–268.6)	0.206
Granulocytes (/µL)	456.0 (0.0–10,510)	162.0 (0.0–50,300.0)	0.005 *
Hypotension	23 (9.9)	19 (6.6)	0.180
Outpatient onset	33 (14.2)	60 (21.0)	0.044 *
Diabetes mellitus	19 (8.2)	39 (13.6)	0.049 *
COPD	1 (0.4)	14 (4.9)	0.002 *
Medical device			
Central venous catheter	107 (45.9)	122 (42.7)	0.456
Ventilator	5 (2.1)	5 (1.7)	0.743
Indwelling urinary catheter	72 (30.9)	58 (20.3)	0.005 *
FUO	130 (55.8)	129 (45.1)	0.015 *
MDI	45 (19.3)	61 (21.3)	0.571
CDI	58 (24.9)	96 (33.6)	0.031 *
Hematologic disease			
Acute myeloid leukemia	55 (23.6)	118 (41.3)	<0.001 *
Myelodysplastic syndrome	27 (11.6)	35 (12.2)	0.820
Acute lymphocytic leukemia	11 (4.7)	12 (4.2)	0.772
Malignant lymphoma	82 (35.2)	73 (25.5)	0.017 *
Multiple myeloma	28 (12.0)	14 (4.9)	0.003 *
Aplastic anemia	9 (3.9)	16 (5.6)	0.359
Chemotherapy within 3 weeks	151 (64.8)	190 (66.4)	0.698
AHSCT	19 (8.2)	17 (5.9)	0.324
Blood culture (two sets) before starting antibiotics	155 (66.5)	209 (73.1)	0.105
Blood culture positivity	54 (23.2)	71 (24.8)	0.662
ESBL and AmpC	16 (6.9)	37 (68.9)	0.023 *

Number (%) or median (interquartile range). Abbreviations are as follows: AST, antimicrobial stewardship team; eGFR, estimated glomerular filtration rate; COPD, chronic obstructive pulmonary disease; FUO, fever of unknown origin; MDI, microbial documented infection; CDI, clinical documented infection; AHSCT, autologous hematopoietic stem cell transplantation; ESBL, Extended spectrum β-lactamases. * *p* < 0.05.

**Table 5:** Multivariable logistic regression of long-term carbapenem therapy (>8 days).

Variable	β	Odds Ratio	95% Confidence Interval	*p*-Value
Age	−0.014	0.986	0.973–1.001	0.059
Granulocytes	0.000	1.000	1.000–1.000	0.127
Outpatient onset	0.533	1.704	1.030–2.818	0.038 *
Diabetes mellitus	0.423	1.527	0.804–2.897	0.196
COPD	2.132	8.436	1.067–66.667	0.043 *
Indwelling urinary catheter	−0.406	0.666	0.421–1.056	0.084
FUO	−0.257	0.773	0.461–1.296	0.329
CDI	0.281	1.325	0.750–2.341	0.333
Acute myeloid leukemia	0.539	1.715	1.057–2.782	0.029 *
Malignant lymphoma	−0.342	0.710	0.438–1.151	0.165
Multiple myeloma	−1.010	0.364	0.168–0.792	0.011 *
ESBL and AmpC	1.036	2.819	1.382–5.750	0.004 *

No. (%) or median (interquartile range). Abbreviations are as follows: COPD, chronic obstructive pulmonary disease; FUO, fever of unknown origin; CDI, clinical documented infection; ESBL, extended spectrum β-lactamases. * *p* < 0.05.

## Data Availability

Not applicable.

## Supplementary Materials

The following supporting information can be downloaded at: https://www.mdpi.com/article/10.3390/antibiotics12020302/s1: Table S1: Clinical data about the disease, medication, or medical device in the pre- and post-AST periods. Table S2: Biological changes or carbapenem perceptions in the pre- and post-AST periods. Table S3: Differences in short- and long-term (>8 days) carbapenem therapies related to demographics, infection disease, or renal function.

## References

[1] Freifeld A.G., Bow E.J., Sepkowitz K.A., Boeckh M.J., Ito J.I., Mullen C.A., Raad I.I., Rolston K.V., Young J.H., Wingard J.R. (2011). Clinical practice guideline for the use of antimicrobial agents in neutropenic patients with cancer: 2010 update by the infectious diseases society of America. Clin. Infect. Dis..

[2] Kuderer N.M., Dale D.C., Crawford J., Cosler L.E., Lyman G.H. (2006). Mortality, morbidity, and cost associated with febrile neutropenia in adult cancer patients. Cancer.

[3] Averbuch D., Orasch C., Cordonnier C., Livermore D.M., Mikulska M., Viscoli C., Gyssens I.C., Kern W.V., Klyasova G., Marchetti O. (2013). ECIL4, a joint venture of EBMT, EORTC, ICHS, ESGICH/ESCMID and ELN. European guidelines for empirical antibacterial therapy for febrile neutropenic patients in the era of growing resistance: Summary of the 2011. Haematol. 4th Eur Conf. Infect. Leuk..

[4] Link H., Böhme A., Cornely O.A., Höffken K., Kellner O., Kern W.V., Mahlberg R., Maschmeyer G., Nowrousian M.R., Ostermann H. (2003). Antimicrobial therapy of unexplained fever in neutropenic patients—Guidelines of the Infectious Diseases Working Party (AGIHO) of the German Society of Hematology and Oncology (DGHO), Study Group Interventional Therapy of Unexplained Fever, Arbeitsgemeinschaft Supportivmassnahmen in der Onkologie (ASO) of the Deutsche Krebsgesellschaft (DKG-German Cancer Society). Ann. Hematol..

[5] Villafuerte-Gutierrez P., Villalon L., Losa J.E., Henriquez-Camacho C. (2014). Treatment of febrile neutropenia and prophylaxis in hematologic malignancies: A critical review and update. Adv. Hematol..

[6] Dellit T.H., Owens R.C., McGowan J.E., Gerding D.N., Weinstein R.A., Burke J.P., Huskins W.C., Paterson D.L., Fishman N.O., Carpenter C.F. (2007). Infectious Diseases Society of America and the Society for Healthcare Epidemiology of America Guidelines for Developing an Institutional Program to Enhance Antimicrobial Stewardship. Clin. Infect. Dis..

[7] Wilson A.P.R. (2017). Sparing carbapenem usage. J. Antimicrob. Chemother..

[8] Hayden M.K., Won S.Y. (2018). Carbapenem-sparing therapy for extended-Spectrum β-lactamase-producing E coli and Klebsiella pneumoniae bloodstream infection: The search continues. JAMA.

[9] Seo S.K., Lo K., Abbo L.M. (2016). Current state of antimicrobial stewardship at Solid Organ and Hematopoietic Cell Transplant Centers in The United States. Infect. Control Hosp. Epidemiol..

[10] Webb B.J., Majers J., Healy R., Jones P.B., Butler A.M., Snow G., Forsyth S., Lopansri B.K., Ford C.D., Hoda D. (2020). Antimicrobial stewardship in a hematological malignancy unit: Carbapenem reduction and decreased vancomycin-resistant enterococcus infection. Clin. Infect. Dis..

[11] la Martire G.L., Robin C., Oubaya N., Lepeule R., Beckerich F., Leclerc M., Barhoumi W., Toma A., Pautas C., Maury S. (2018). De-escalation and discontinuation strategies in high-risk neutropenic patients: An interrupted time series analyses of antimicrobial consumption and impact on outcome. Eur. J. Clin. Microbiol. Infect. Dis..

[12] Gedik H. (2017). Antibiotic resistance status and its costs in hematological patients: A two-year analysis. Casp. J. Intern. Med..

[13] O’Horo J.C., Marcelin J.R., Abu Saleh O.M.A., Barwise A.K., Odean P.M., Rivera C.G., Tande A.J., Wilson J.W., Osmon D.R., Tosh P.K. (2019). Standardizing febrile neutropenia management: Antimicrobial stewardship in the hematologic malignancy population. J. Oncol. Pr..

[14] Nørgaard M., Larsson H., Pedersen G., Schønheyder H.C., Sørensen H.T. (2006). Risk of bacteraemia and mortality in patients with haematological malignancies. Clin. Microbiol. Infect..

[15] Guisado-Gil A.B., Aguilar-Guisado M., Peñalva G., Lepe J.A., Espigado I., Rodríguez-Arbolí E., González-Campos J., Rodríguez-Torres N., Montero-Cuadrado M.I., Falantes-González J.F. (2021). Long-term impact of an educational antimicrobial stewardship program on management of patients with hematological diseases. Antibiotics.

[16] Madran B., Keske Ş., Tokça G., Dönmez E., Ferhanoğlu B., Çetiner M., Mandel N.M., Ergönül Ö. (2018). Implementation of an antimicrobial stewardship program for patients with febrile neutropenia. Am. J. Infect. Control.

[17] Mardani M., Abolghasemi S., Shabani S. (2020). Impact of an antimicrobial stewardship program in the antimicrobial-resistant and prevalence of Clostridioides difficile infection and amount of antimicrobial consumed in cancer patients. BMC Res. Notes.

[18] Puerta-Alcalde P., Cardozo C., Suárez-Lledó M., Rodríguez-Núñez O., Morata L., Fehér C., Marco F., Del Río A.D., Martínez J.A., Mensa J. (2019). Current time-to-positivity of blood cultures in febrile neutropenia: A tool to be used in stewardship de-escalation strategies. Clin. Microbiol. Infect..

[19] Le Clech L., Talarmin J.P., Couturier M.A., Ianotto J.C., Nicol C., Le Calloch R., Dos Santos S., Hutin P., Tandé D., Cogulet V. (2018). Early discontinuation of empirical antibacterial therapy in febrile neutropenia: The ANTIBIOSTOP study. Infect. Dis..

[20] Haynes R.B., Devereaux P.J., Guyatt G.H. (2002). Physicians’ and patients’ choices in evidence based practice. BMJ.

[21] Djulbegovic B., Guyatt G.H. (2017). Progress in evidence-based medicine: A quarter century on. Lancet.

[22] Song X., Peng Y., Wang X., Chen Y., Jin L., Yang T., Qian M., Ni W., Tong X., Lan J. (2018). Incidence, survival, and risk factors for adults with acute myeloid leukemia not otherwise specified and acute myeloid leukemia with recurrent genetic abnormalities: Analysis of the surveillance, epidemiology, and end results (SEER) Database, 2001–2013. Acta Haematol..

[23] De Lima M.C., Da Silva D.B., Freund A.P.F., Dacoregio J.S., Costa T.J., Costa I., Faraco D., Silva M.L. (2016). Acute myeloid leukemia: Analysis of epidemiological profile and survival rate. J. Pediatr..

[24] Kumar S.K., Dispenzieri A., Lacy M.Q., Gertz M.A., Buadi F.K., Pandey S., Kapoor P., Dingli D., Hayman S.R., Leung N. (2014). Continued improvement in survival in multiple myeloma: Changes in early mortality and outcomes in older patients. Leukemia.

[25] Landgren O., Iskander K. (2017). Modern multiple myeloma therapy: Deep, sustained treatment response and good clinical outcomes. J. Intern. Med..

[26] Niessen F.A., van Mourik M.S.M., Bruns A.H.W., Raijmakers R.A.P., de Groot M.C.H., van der Bruggen T. (2020). Early discontinuation of empirical antibiotic treatment in neutropenic patients with acute myeloid leukaemia and high-risk myelodysplastic syndrome. Antimicrob. Resist. Infect. Control.

[27] Klastersky J., Paesmans M., Rubenstein E.B., Boyer M., Elting L., Feld R., Gallagher J., Herrstedt J., Rapoport B., Rolston K. (2000). The Multinational Association for Supportive Care in Cancer Risk Index: A Multinational Scoring System for Identifying Low-Risk Febrile Neutropenic Cancer Patients. J. Clin. Oncol..

[28] Alberta Provincial Tumour Teams (2014). Management of febrile neutropenia in adult cancer patients. Albertha. Health Serv..

[29] Sethi S., Murphy T.F. (2008). Infection in the pathogenesis and course of chronic obstructive pulmonary disease. N. Engl. J. Med..

[30] Havey T.C., Fowler R.A., Daneman N. (2011). Duration of antibiotic therapy for bacteremia: A systematic review and meta-analysis. Crit. Care.

[31] Tamma P.D., Aitken S.L., Bonomo R.A., Mathers A.J., van Duin D., Clancy C.J. (2022). Infectious Diseases Society of America Guidance on the Treatment of Extended-Spectrum β-lactamase Producing Enterobacterales (ESBL-E), Carbapenem-Resistant Enterobacterales (CRE), and Pseudomonas aeruginosa with Difficult-to-Treat Resistance. Clin. Infect. Dis..

[32] Komagamine J., Yabuki T., Hiraiwa T. (2019). A trend in prevalence of antimicrobial use and appropriateness of antimicrobial therapy in an acute care hospital from 2018 to 2019: Repeated prevalence surveys in Japan. BMC Res. Notes.

[33] ATC/DDD Index 2022. https://www.whocc.no/atc_ddd_index/.

[34] Matsuo S., Imai E., Horio M., Yasuda Y., Tomita K., Nitta K., Yamagata K., Tomino Y., Yokoyama H., Hishida A. (2009). Comparative Study Revised Equations for Estimated GFR from Serum Creatinine in Japan. Am. J. Kidney Dis..

[35] Kidney Disease: Improving Global Outcomes (KDIGO) Glomerular Diseases Work Group (2021). KDIGO 2021 Clinical Practice Guideline for the Management of Glomerular Diseases. Kidney Int..

[36] Suzuki A., Maeda M., Yokoe T., Hashiguchi M., Togashi M., Ishino K. (2021). Impact of the multidisciplinary antimicrobial stewardship team intervention focusing on carbapenem de-escalation: A single-centre and interrupted time series analysis. Int. J. Clin. Pract..

